# Analytical interference of intravascular contrast agents with clinical laboratory tests: a joint guideline by the ESUR Contrast Media Safety Committee and the Preanalytical Phase Working Group of the EFLM Science Committee

**DOI:** 10.1007/s00330-023-10411-x

**Published:** 2023-12-07

**Authors:** Aart J. van der Molen, Johannes G. Krabbe, Ilona A. Dekkers, Remy W. F. Geenen, Marie-France Bellin, Michele Bertolotto, Torkel B. Brismar, Janne Cadamuro, Jean-Michel Correas, Gertraud Heinz-Peer, Michel R. Langlois, Andreas H. Mahnken, Tomris Ozben, Carlo C. Quattrocchi, Alexander Radbruch, Peter Reimer, Giles Roditi, Laura Romanini, Carmen Sebastià, Ana-Maria Simundic, Fulvio Stacul, Olivier Clement

**Affiliations:** 1 Department of Radiology, Leiden University Medical Center, Leiden, The Netherlands; 2 Department of Clinical Chemistry and Laboratory Medicine, Medisch Spectrum Twente, Enschede, The Netherlands; 3 Department of Radiology, Northwest Clinics, Alkmaar, The Netherlands; 4 University Paris-Saclay, AP-HP, University Hospital Bicêtre, Service de Radiologie, BioMaps, Le Kremlin-Bicêtre, France; 5 Department of Radiology, University Hospital Trieste, Trieste, Italy; 6 Department of Clinical Science, Intervention and Technology, Unit of Radiology, Karolinska Institutet and Department of Radiology, Karolinska University Hospital in Huddinge, Stockholm, Sweden; 7 Department of Laboratory Medicine, Paracelsus Medical University, Salzburg, Austria; 8 Université de Paris, AP-HP, Groupe Hospitalier Necker, DMU Imagina, Service de Radiologie, Paris, France; 9 Department of Radiology, Landesklinikum St Pölten, St Pölten, Austria; 10 Department of Laboratory Medicine, AZ St.-Jan Hospital, Brugge, Belgium; 11 Department of Radiology, Marburg University Hospital, Marburg, Germany; 12 Department of Clinical Biochemistry, Faculty of Medicine, Akdeniz University, Antalya, Turkey; 13 Centre for Medical Sciences - CISMed, University of Trento, Trento, Italy; 14 Clinic for Diagnostic and Interventional Neuroradiology, University Clinic Bonn, and German Center for Neurodegenerative Diseases, DZNE, Bonn, Germany; 15 Department of Radiology, Institute for Diagnostic and Interventional Radiology, Klinikum Karlsruhe, Karlsruhe, Germany; 16 Department of Radiology, Glasgow Royal Infirmary, Glasgow, UK; 17 Department of Radiology, ASST Cremona, Cremona, Italy; 18 Department of Radiology, Hospital Clinic de Barcelona, Barcelona, Spain; 19 Department of Medical Laboratory Diagnostics, Clinical Hospital Sveti Duh, Zagreb University, Zagreb, Croatia; 20 Department of Radiology, Ospedale Maggiore, Trieste, Italy; 21 Université de Paris, AP-HP, Hôpital Européen Georges Pompidou, DMU Imagina, Service de Radiologie, 20 Rue LeBlanc, F-75015 Paris, France

**Keywords:** Analytical chemistry, Clinical laboratory techniques, Contrast agents, Diagnostic error, Clinical practice guideline

## Abstract

**Abstract:**

The Contrast Media Safety Committee of the European Society of Urogenital Radiology has, together with the Preanalytical Phase Working Group of the EFLM Science Committee, reviewed the literature and updated its recommendations to increase awareness and provide insight into these interferences.

**Clinical relevance statement:**

Contrast Media may interfere with clinical laboratory tests. Awareness of potential interference may prevent unwanted misdiagnosis.

**Key Points:**

*• Contrast Media may interfere with clinical laboratory tests; therefore awareness of potential interference may prevent unwanted misdiagnosis.*

*• Clinical Laboratory tests should be performed prior to radiological imaging with contrast media or alternatively, blood or urine collection should be delayed, depending on kidney function.*

## Introduction

Radiological imaging with, or without, contrast agents (CA) and laboratory tests are commonly used in the diagnosis and monitoring of patients. In terms of efficient patient work-up, these tools are often performed concomitantly and/or serially. However, the presence of iodine-based contrast media (ICM) and gadolinium-based contrast agents (GBCA) may interfere with several clinical laboratory tests. Awareness of these inferences is potentially important since they may pose a potential threat by misinterpretation and/or incorrect monitoring of patients, denying or delaying their treatment or initiating/continuing potentially harmful treatment [1]. These clinically relevant interferences are specific for the contrast media administered as well as for the specific technique/method used for the analysis of the biomarker [2]. However, the effect of CM on clinical laboratory tests has not been studied systematically nor extensively, therefore relying on the limited available evidence. Here we present an overview of analytical interference of intravascularly administrated, clinically approved ICM or GBCA on commonly used clinical laboratory tests as well as an expert/consensus opinion about when and how to use blood and/or urine analysis during or after radiological imaging with CA.

## Methodology (materials and methods)

For this narrative review, the literature was analyzed using PubMed and Embase databases from January 1990 until May 2022. Multiple repetitive searches with search criteria including synonyms of “contrast agents”, “gadolinium-based”, “iodine-based”, “analytical interference” and “laboratory interference” were performed for all clinically approved iodine-based and gadolinium-based contrast agents, with languages limited to English and German. After removal of duplications, the initial search resulted in 384 studies. These were subsequently evaluated for suitability by two experienced reviewers (J.K., A.J.v.d.M.) in two stages, first on title and abstract, and then on full text. Results from cross-referencing were added where appropriate. In total, 29 studies were included in the final review. Papers were selected based on clinically relevant interference of intravascularly administrated, clinically approved contrast agents on biomarkers. Moreover, papers addressing physiological effect of CA on biomarkers and papers addressing CA or analytical methods currently not in use (anymore) in clinical practice or analytical interference solely reported in animals, were excluded. Also excluded were non-vascular (oral, rectal, intracavitary, etc.) administration of these contrast agents, the use of chromophores for diagnostic use, and the potential of liver-specific GBCA for functional imaging. The concept guideline manuscript was discussed and agreed upon in revised form at a meeting of the members of the Contrast Media Safety Committee (CMSC) of the European Society of Urogenital Radiology (ESUR) in June 2022 in Paris (France), and subsequently endorsed by the Preanalytical Phase Working Group of the Science Committee of the European Federation of Clinical Chemistry and Laboratory Medicine (EFLM).

## Results

Several studies have demonstrated interference by CA on a wide variety of clinical laboratory tests. Depending on the CM and analytical method used, a positive, negative or no bias is observed. These interferences can be divided based on the type of CA, either ICM or GBCA.

### Iodine-based contrast media

The effect of ICM on clinical assays has not been studied systematically or extensively. Depending on the method and ICM used, interference may be clinically relevant [3]. M-protein analysis is paramount in the diagnosis and monitoring of monoclonal gammopathy [4]. Several studies report the interference of ICM on the spectrophotometric detection of monoclonal protein analysis in urine and blood by capillary zone electrophoresis with spectrophotometric detection (CZE-UV) [5]. ICM absorbs UV light at a similar wavelength as the peptide bonds in m-proteins, thereby mimicking the presence of (M-) proteins in the commonly used CZE analysis with UV detection. In contrast, Capaldo and co-workers [6] demonstrated that the opposite may also occur, i.e. masking of an M-protein peak. In the M-protein analysis by CZE-UV, a duplication in the beta-2 fraction, which was at first assigned to ICM (iomeprol) interference and the beta-1 fraction, did not display any M-protein peak. These specific cases demonstrate that ICMs may cause incorrect detection of an M-protein, resulting in unnecessary diagnostics and/or treatment or on the other hand missing an M-protein thereby delaying treatment.

Otnes and co-workers investigated the analytical interference of two specific ICM, iodixanol and iomeprol [2] in vitro. They reported in the high, but clinically relevant, concentration range of the ICM, either a positive bias (colorimetric calcium assay) or a negative bias, i.e. colorimetric iron, magnesium, and zinc assay as well as in the direct potentiometric sodium assay. Other assays did not show any interference with both ICM.

ICM can affect immunoassays differently, depending on the manufacturer. Such an example is when evaluating cardiac Troponin-I in patients undergoing coronary angiography. When evaluating two different assays, the Opus Magnum (Behring Diagnostics) and the Access (Beckman Coulter, Inc.) using 12 different ICM, Lin et al [7] showed that the outcome of the Opus system was affected when performed directly after the coronary angiography procedure, but not after 30 min in patients with normal kidney function. In patients with reduced kidney function, the interference lasted longer. The access assay did not show any interference.

An interference by iohexol on endocrine immunoassays was observed by Loh and co-workers in in-vitro experiments [8]. They reported that soon after contrast administration, iohexol may affect follicle-stimulating hormone (FSH), luteinizing hormone (LH), plasma renin activity (PRA) and thyroid stimulating hormone (TSH) measurements by different manufacturers, either over- or underestimating the true value. The interference on immunoassays may be explained by either the presence of an unidentified antigenic site on the contrast medium molecule blocking or cross-reacting with antibodies of the immunoassay, dilutional effects due to the high osmolar aspects of iohexol and/or, as described before, due to spectrophotometric aspects of the ICM, interfering with UV-detection. No other ICM were studied by Loh et al and most of the interference effects were seen only at very high iohexol concentrations, which is very uncommon in clinical practice.

Next to the photometric aspects of ICM, the analysis of the specific gravity of urine uses the refractive index. A higher refractive index due to the presence of the ICM in urine may produce false results [9–11].

Besides interference in laboratory testing, sample integrity and quality may be impacted [12].

Since the density of blood is altered due to the presence of ICM in the blood, the gel cell separator characteristics may be altered, resulting in incorrect plasma or serum collection [13–15] and thereby causing mechanical problems by clogging sample needles in the routine platforms.

Table 1 shows demonstrated ICM interference on clinical laboratory tests.

**Table 1:** Clinical and/or analytical significant biomarker interference of specific ICM

Iodine-based contrast media				
Analyte	Method/technique	Name ICM	Observed Interference (bias)*	Reference
Albumin	Colorimetric assay	Iodixanol	↑	[2]
Aldosterone	Radioimmunoassay with I^125^-tracer	Iohexol	↓	[8]
Bicarbonate	Enzymatic assay	Iomeprol, iodixanol	↓	[2]
Calcium	Colorimetric assay	Iomeprol, iodixanol	↑	[2]
Chloride	Ion selective electrode	Iohexol	↓	[16]
Cortisol	Immunoassay with spectrophotometric detection	Iohexol	↑	[8]
C-peptide	Immunoassay with spectrophotometric detection	Iohexol	↓	[8]
Erythrocytes in urine	Fluorescence flow cytometry	Iomeprol	↑	[10]
Follicle Stimulating Hormone	Immunoassay with spectrophotometric detection	Iohexol	↓	[8]
Insulin	Immunoassay with spectrophotometric detection	Iohexol	↓	[8]
Iron	Colorimetric assay	Iodixanol	↑	[2]
LDH	Enzymatic assay	Iodixanol	↓	[2]
Leukocytes in urine	Fluorescence flow cytometry	Iomeprol	↑	[10]
Luteinizing Hormone	Immunoassay with spectrophotometric detection	Iohexol	↓	[8]
Magnesium	Colorimetric assay	Iomeprol	↓	[2]
M-proteins	CZE-UV	Iomeprol, iohexol, sodium/meglumine amidotrizoate, ioversol, iopromide, iobitridol, iopamidol, sodium ioxitalamate	↑, ↓	[6, 17]
Potassium	Potentiometric assay	Iodixanol, iomeprol	↑	[2]
Renin activity	Radioimmunoassay with I^125^-tracer	Iohexol	↓	[8]
Sodium	Potentiometric assay, Ion selective electrode	Iomeprol, iodixanol, iohexol	↓	[2, 16]
Specific gravity in urine	Refractometry	Iomeprol, iohexol, iodixanol	↑	[9, 10]
Thyroid Stimulating Hormone	Immunoassay with spectrophotometric detection	Iohexol	↓	[8]
Troponin I	Immuno-enzymatic assay	11 ICMs, among them iopromide, ioversol, iohexol	↑	[7]
Zinc	Colorimetric assay	Iodixanol	↓	[2]

^*^↓ Negative interference (underestimation), ↑ positive interference (overestimation)

*N.B. interference may be manufacturer/analyser specific. For detailed information see references*

### Gadolinium-based contrast agents

Since their clinical approval and introduction in 1988, GBCAs have been administered in 750 million standard doses (Bayer Healthcare, estimated from multiple internal and external sources). Several interferences on laboratory tests have been described, ranging from commonly used laboratory tests [12] to more specialized laboratory tests [18]. The probably most clinically relevant interference is the interference of GBCAs on serum calcium measurement by specific colorimetric assays. Gadodiamide [19–22] and gadoversetamide [7] are the GBCAs most frequently reported to interfere. Those are no longer on the market, but it should be noted that the interference has been shown to occur irrespective of the molecular configuration of the chelate (i.e. linear or macrocyclic and ionic or non-ionic) [20], although the largest interference was observed on GBCAs with a linear molecular configuration of the ligand [23]. This interference has not been observed with other serum calcium measurement tests, e.g. Ca-specific electrode, atomic absorption or mass spectrometry.

In an in vitro study, Proctor and co-workers [24] investigated the analytical interference of four GBCAs on multiple analytes and multiple analysers. They demonstrated that depending on the specific GBCA a positive and negative analytical interference is observed, which is most prominent in Angiotensin Converting Enzyme (ACE), calcium, iron, total iron binding capacity (TIBC), magnesium and zinc. Mechanistically, all the affected analytes are either endogenous divalent cations or somehow use divalent cations in the reaction of the laboratory test. Gd^3+^ can interact with the analyte of interest (e.g. transmetallation), thereby potentially interrupting the analytical process, or in colorimetric assays by binding with the chromophore [23]. In an in-vitro experiment, Otnes and co-workers demonstrated a similar interference by the GBCAs gadoxetate disodium, gadoterate meglumine, and gadobutrol on iron and zinc (negative bias) assays. Other 29 clinical tests did not display any clinically relevant interference by these GBCAs [2].

In the field of trace elements and heavy metals, inductively coupled plasma mass spectrometry (ICP-MS) is the golden standard. Gd^3+^ may interfere also with this technique in multiple ways, i.e. space-charge effects, interference in the mass spectrometric analysis by double-charged ions and polyatomic interference [18]. The latter can be circumvented by applying the correct analytical technique. Especially the analysis of selenium by ICP-MS may be complicated by the presence of ^156^Gd due to similar mass-to-charge ratios. Gd ions may also interfere with the ionization process, suppressing ions of analytes, e.g. trace elements or (toxic) heavy metals and internal standards used.

An increase in urinary Zn and Cu concentration was seen, especially with gadodiamide [25]. This increase is, as the authors hypothesized, possibly related to in vivo transmetallation and not to a true analytical interference, and was therefore excluded.

Table 2 shows described GBCA interference on clinical laboratory tests.

**Table 2:** Clinical and/or analytical significant biomarker interference of specific GBCA

Gadolinium-based contrast agents				
Analyte	Method/technique	Name GBCA	Observed interference (bias)*	Reference
ACE	Colorimetric enzymatic reaction	Gadodiamide, gadoversetamide	↓	[24]
Calcium	Several colorimetric assays	Gadodiamide, gadoversetamide	↓	[23, 24]
Iron	Colorimetric assay	Gadodiamide, gadoversetamide, gadopentetate dimeglumine, gadoxetate disodium	↓,↑	[2, 24]
Magnesium		Gadodiamide, gadoversetamide	↓,↑	[24]
Selenium	ICP-MS	Not specified	↑	[26]
TIBC	Colorimetric assay	Gadodiamide, gadoversetamide	↑	[24]
Troponin I	Immuno-enzymatic assay	Gadopentetate dimeglumine	↑	[7]
Zinc	Colorimetric assay	Gadodiamide, gadoversetamide, gadopentetate dimeglumine, gadoteridol, gadoxetate disodium	↓	[2, 24]

↓ Negative interference (underestimation), ↑ positive interference (overestimation)

*Interference may be manufacturer/analyser specific. For detailed information see references*

*Gadodiamide and gadoversetamide are currently not on the market in the EU*

### Clearance of contrast media

Most studies on iodine-based contrast media (ICM) employ an open, 2-compartment model for pharmacokinetic analyses. The first compartment is the plasma in which the molecules are being diluted and the second compartment is the extracellular volume, excluding the brain (due to the blood-brain barrier). The plasma concentration decays by distribution of the ICM from plasma to the extracellular volume (distribution phase, rate constant α), and by elimination of the CM from plasma to urine by renal excretion (elimination phase, rate constant β).

Biodistribution studies have suggested that an open 3-compartment model may better fit the pharmacokinetic data of GBCA. The second and third compartments are the extravascular extracellular spaces of rapidly and slowly equilibrating tissues (storage compartment (of unknown exact composition)). Apart from the distribution phase and the rapid (renal) elimination phase, there is a slow residual excretion phase that is species-independent and whose rate constant γ is closely related to the thermodynamic stability of the specific GBCA molecule [27, 28].

Contrast media are eliminated through glomerular filtration. In addition, the liver-specific GBCAs have partial hepatic excretion of up to 50% of the intravascular administered dose. With a normal glomerular filtration rate (GFR), 90 mL/min/1.73 m^2^, the half-life in plasma is about 2 h, roughly for both ICM and GBCA, although in normal renal function, half-life is on average somewhat shorter for GBCA. In patients with advanced renal function loss with a GFR < 30 mL/min/1.73 m^2^, the half-life may increase up to 30 h [28]. Near-complete elimination to 1.5% of the original concentration occurs after 6 elimination half-lives. Thus, to avoid interference from contrast media, sampling should be delayed as outlined in Table 3, depending on the renal function of the patient.

**Table 3:** Recommendations of delay in blood or urine collection after administration of contrast media, based on kinetic and clearance information [28]

Kidney Function	Delay blood collection by:	Delay urine collection by:
eGFR > 60 mL/min/1.73 m^2^	At least 4 h and optimally 12 h after administration of the contrast medium	At least 24 h after administration of the contrast medium
eGFR 30–60 mL/min/1.73 m^2^	At least 16 h and optimally 48 h after administration of the contrast medium	At least 48 h after administration of the contrast medium
eGFR < 30 mL/min/1.73 m^2^	At least 2.5 days (60 h) and optimally 7 days (168 h) after administration of the contrast medium	At least 7 days (168 h) after administration of the contrast medium

## Discussion

Several reports have demonstrated clinically relevant CM interference on clinical laboratory tests with potential hazardous adverse outcomes. Interference may rely on the analytical method used, including colorimetric assays, immunoassays and even mass spectrometric techniques as well as on the CM used. Moreover, depending on the specific assay, an over- or underestimation has been reported. For colorimetric assays, the potential bias could be either absent, positive, or negative. When measuring calcium levels, the interference from ICM can be avoided by using ICP-MS instead. However, GBCA may interfere with ICP-MS analysis, depending on the biomarker of interest.

Current recommendations rely mainly on CM elimination. ESUR [3, 28, 29], for instance, currently recommends performing blood and urine clinical tests prior to administration of the contrast medium, to circumvent interference and incorrect assessment of the patient. Post-imaging non-emergency blood and urine analysis should be delayed until the CM concentration in blood and/or urine is not present anymore. In emergency testing, blood and urine analysis can be performed, though clinicians and laboratory specialists should be aware of the potential interference of CM. Moreover, automatic drug-laboratory test interaction alerts may further help in this awareness [30]. As with all laboratory tests, the test results should be interpreted in relationship with the patient’s medical history and clinical examination.

The scope of this study was focused on the analytical interference of clinical laboratory tests. CA may also influence the physiological status, resulting in altered biomarker concentrations such as the effect of CA on blood coagulation or osmotic diuresis.

## Conclusion

Analytical interference of contrast agents on (routine) clinical laboratory tests can potentially be hazardous by causing misinterpretation of results with subsequent incorrect diagnosis and/or undesirable treatment decisions. Ideally, these clinical laboratory tests are performed prior to radiological imaging with contrast media. If this is not possible, the advice is to delay the blood withdrawal or urine collection. Simple guidelines are proposed (Table 3).

In general, awareness of potential interference may prevent unwanted misdiagnosis.

## Abbreviations

ACE: Angiotensin converting enzyme
CM: Contrast medium/media
CMSC: Contrast Media Safety Committee
CZE: Capillary zone electrophoresis
EFLM: European Federation of Clinical Chemistry and Laboratory Medicine
ESUR: European Society of Urogenital Radiology
FSH: Follicle stimulating hormone
GBCA: Gadolinium-based contrast agent/agents
ICM: Iodine-based contrast medium/media
ICP-MS: Inductively coupled plasma mass spectrometry
LH: Luteinizing hormone
PRA: Plasma renin activity
TIBC: Total iron binding capacity
TSH: Thyroid stimulating hormone
UV: Ultraviolet

## Declarations

### Guarantor

The scientific guarantor of this publication is Olivier Clément.

### Statistics and biometry

No complex statistical methods were necessary for this paper.

### Methodology

• Retrospective literature review
